# Investigation on Chemical Composition, Antioxidant, Antifungal and Herbicidal Activities of Volatile Constituents from *Deverra tortuosa* (Desf.)

**DOI:** 10.3390/plants12132556

**Published:** 2023-07-05

**Authors:** Marwa Khammassi, Flavio Polito, Oumayma Kochti, Habiba Kouki, Mouna Souihi, Sana Khedhri, Lamia Hamrouni, Yassine Mabrouk, Ismail Amri, Vincenzo De Feo

**Affiliations:** 1 Laboratory of Management and Valorization of Forest Resources, National Institute of Researches on Rural Engineering, Water and Forests, P.B. 10, Ariana 2080, Tunisia; sanakhedhrii@gmail.com (S.K.); hamrounilam@yahoo.fr (L.H.); amri_amri@live.fr (I.A.); 2 Department of Pharmacy, University of Salerno, Via San Giovanni Paolo II, 132, 84084 Fisciano, Italy; fpolito@unisa.it (F.P.); defeo@unisa.it (V.D.F.); 3 Laboratory of Biotechnology and Nuclear Technology, National Center of Nuclear Science and Technology, Sidi Thabet, B.P. 72, Ariana 2020, Tunisia; kochtimayma@gmail.com (O.K.); habibakouki96@gmail.com (H.K.); souihimouna@gmail.com (M.S.); mabrouk.yassine@cnstn.rnrt.tn (Y.M.); 4 Institute of Food Science, CNR-ISA, Via Roma, 64, 83100 Avellino, Italy

**Keywords:** *Deverra tortuosa*, essential oils, antioxidant activity, phytotoxicity, antifungal activity

## Abstract

This study aims to analyze the chemical composition of the essential oils (EOs) obtained from stems and umbels of *D. tortuosa* as well the assessment of their biological activity. EOs were extracted by hydrodistillation and analyzed by gas chromatography coupled to mass spectrometry (GC/MS). The antioxidant properties were determined by DPPH and ABTS assays. The phytotoxic potential was assessed against dicots weeds (*Sinapis arvensis* and *Trifolium campestre*), monocots weeds (*Lolium rigidum*) and the crop *Lepidium sativum*. The antifungal activity was evaluated against four target phytopathogenic fungal strains. High diversity of compounds was detected in *D. tortuosa* Eos, varying among plant parts and consisting mainly of *α*-pinene (24.47–28.56%), sabinene (16.2–18.6%), *α*-phellandrene (6.3–11.7%) and *cis*-ocimene (5.28–7.85%). *D. tortuosa* EOs exhibited remarkable antioxidant activity, as well as interesting variable antifungal activities depending on the dose and fungi strain. The herbicidal activity of EOs showed significant efficacy on the inhibition of germination and seedling growth of all tested herbs. These results suggest that the EOs of *Deverra tortuosa* represent a valuable source of antioxidant, antifungal and phytotoxic metabolites and could be potential candidates for pest management, contributing to the promotion of sustainable agriculture.

## 1. Introduction

Appreciation and utilization of natural bioactive substances in aromatic plants have increased in recent years [1]. It is known that aromatic plants are considered as alternatives for microbial resistance and as a useful avenue for safe pest control and management [1,2]. Currently, pest-related problems are largely controlled through the application of chemical pesticides [3]. This generalized use makes it possible to control the majority of pests, but it induces generally undesirable side effects whose acuity is increasing, such as influence on the microflora of the soil, development of biotypes resistant to pesticides, contamination of the environment and generation of harmful effects on human health [4]. In addition to the changes that affect our environment and climate, they also affect the whole dynamic of life by disturbing the ecosystems and the species that constitute them. These effects have an impact, in particular, on the interactions between species and their environment [5,6]. These interactions are crucial for the maintenance of ecosystems where chemical mediation plays a preponderant place. Hence, major technological advances in the field of the chemistry of natural substances have become essential to respond to current issues.

In this context, allelopathy, defined as the direct or indirect and positive or negative effects of a plant on another through the production of chemical compounds released into the environment, is a part of the interferences between plants and their biotopes via chemical mediators. Indeed, many plant species mainly synthesize secondary metabolites that are involved in allelopathic interactions [7,8]. These molecules may inhibit the germination and growth of plants growing in their vicinity and are also involved in the defense and inhibition of fungi, bacteria, harmful insects and even herbivores [7]. For that, these metabolites are called allelochemicals and possess several biological activities: herbicide, antimicrobial, insecticide and nematicide [9,10]. The understanding of these interactions and their modes of action can contribute to the control of the constraints which disturb the qualitative and quantitative production of the fields of culture and allow the development of sustainable agriculture, and especially the protection of the environment and human health by limiting the use of harmful chemicals.

Several families of plants are known for their richness in botanical metabolites, often providing allelopathic potential. The Apiaceae family is well known for its richness in active molecules, and a great diversity in the production of these molecules has been reported in the literature [11,12]. This family is the largest of the order of the Umbellales, commonly known as the parsley or carrot family, which contains 300 genera and more than 3000 species, especially widespread in the northern hemisphere [13].

The genus *Deverra* (Apiaceae) involves thirteen taxa, nine species and four subspecies with distinctive distribution in North Africa and South Africa to Arabian Ecoregions [14]. Actually, some species belonging to this genus, in the form of plant extracts, EOs and infusions of aerial parts, are used in traditional folk medicine [15,16,17,18].

*Deverra tortuosa* (Desf.) DC. is widely distributed in central Tunisia and has various local traditional uses in folk medicine as a remedy for high blood pressure [19,20] and to relieve stomach pain and fight intestinal parasites [21,22]. Additionally, the tender sprouts are employed as an appetite suppressant or added to salads [23]. It is also used as a carminative and treatment for constipation and scorpion stings [20,24]. Some studies reported its biological potentialities and confirmed its widespread uses [25]. In fact, the EO from aerial parts was shown to be anti-tumoral [26]. Furthermore, the EOs of *D. tortuosa* grown in Saudi Arabia showed antioxidant and antifungal activities [27]. In addition, an interesting cytotoxic effect was reported against liver, colon and breast cancer cell lines [28]. Moreover, Guesmi et al. (2017) demonstrated cytotoxic effects against six tumor cell lines [29]. Abd El-Moaty and coworkers showed that aqueous extracts of *D. tortuosa* contain phenolic compounds, coumarins, alkaloids, tannins, proteins and terpenoids [30]. The evaluation of their biological activities revealed their effectiveness by acting as a reducing and capping agent. Phyto-synthesized gold nanoparticles from an aqueous extract have been reported to reduce the level of blood sugar and could be used as potent anti-*Helicobacter pylori* agents [30].

A recent study revealed the allelopathic potential of aqueous extracts of *D. toutuosa* growing in Saudi Arabia on the germination and growth of *Medicago polymorpha* L. seedlings [31]. However, only one study has been carried out on the phytotoxic effects of EOs obtained from *D. toutuosa* against two cultivated species [32]. In fact, the majority of the literature reports are focused on medicinal and pharmaceutical applications [26,27,28,29,30], and the application of molecules from this species in allelopathy and pest management is still limited. On the other hand, this species grows in generally extreme conditions; it is a quintessentially Saharan species. This could explain the ability of this species to adapt and acclimatize to severe ecological conditions, mainly through the production of allelochemicals [18,33]. Therefore, the study of *D. tortuosa* metabolites can be useful for agronomic applications and the search of molecules with herbicidal and antifungal properties.

In this sense, the current study aims to examine the chemical composition and the antioxidant, phytotoxic and antifungal activities of the EOs obtained from stems and umbels of *D. tortuosa*. The phytotoxicity test was carried out against the germination and growth of weeds, dicots (*Sinapis arvensis* and *Trifolium campestre*) and monocots (*Lolium rigidum*), and also one cultivated species, *Lepidium sativum.* The antifungal activity was evaluated against *Fusarium culmorum*, *F. oxysporum* f. sp. *mathioli*, *F. solani* and *F. oxysporum* f. sp. *Lycopersici*.

## 2. Results and Discussion

### 2.1. Yield and Chemical Composition

EOs were obtained from the stems and umbels of *D. tortuosa* with variable yields. Umbels showed the highest yield (1.61 ± 0.15), twice that of stems (0.74 ± 0.1). This variability agrees with recent studies. Indeed, a yield of 0.56% was obtained from the entire plant (stems and umbels mixed) from Egypt [34]. Yields of around 0.31 and 0.85 were also recorded in Lybian plants collected in two different seasons, spring and summer, respectively [35]. In Tunisia, yields vary from 1.1 to 2% depending on the drying of the aerial parts [21,36,37]. The majority of studies are focused on the whole plant; however, the variability according to the umbels and the stems was not described. In general, the Apiaceae family is known for its richness in EOs, as well as the richness and variability of production by the different parts of the plant. Similarly, the differences in yields observed in the current study and those described in the literature are the result of several extrinsic and intrinsic factors.

The study of the chemical composition of the EOs obtained from the stems and umbels of *D. tortuosa* was carried out by GC-FID and GC-MS. Twenty-eight components have been identified, representing 97.14 and 99.49% of the total for stems and umbels, respectively. The composition of the EOs is reported in Table 1.

**Figure 1:**
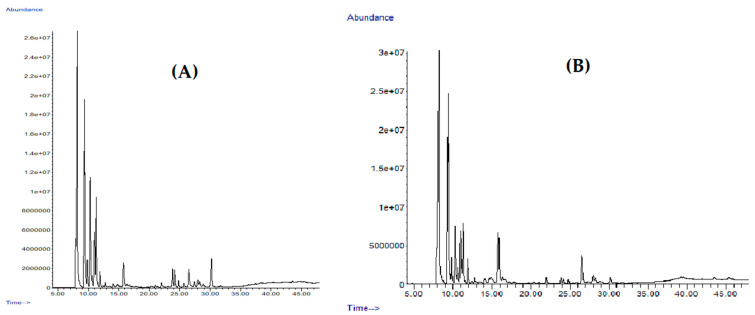
Chromatograms of essential oils from aerial parts of *D. tortuosa* on a HP-5MS column. (**A**): Umbels; (**B**): Stems.

A clear variability of the chemical composition between the EOs from stems and umbels was observed, both in the presence/absence and in the amounts of the components (Figure 1).

**Figure 2:**
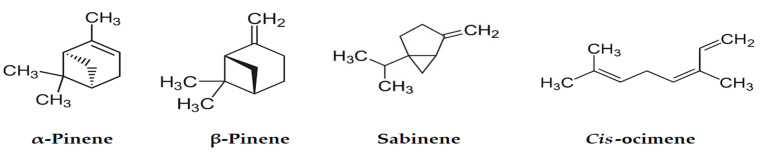
Major hydrocarbon monoterpenes of *D. tortuosa* stems and umbels.

Both EOs were characterized by a richness in hydrocarbon monoterpenes with a rate exceeding 80% of the total. The main components (Figure 2) of this fraction were *α*-pinene (24.2–28.8%), sabinene (16.23–18.67%), *β*-pinene (5.1–6.2%) and *cis*-ocimene (5.28–7.85%) for umbels and stems, respectively, with similar amounts for stems and umbels; however, *α*-phellandrene varied between 6.3 and 11.74% in umbels and stems, respectively.

However, *β*-phellandrene (6.7%) was only detected in the EO from stems, and *p*-cymene (6.77%) was detected only in umbels. *p*-cymene is a monoterpene, naturally present in certain plants, in particular, in Apiaceae species. This compound is known to have several biological activities [42,43] and is an important industrial intermediate used in the synthesis of fungicides, pesticides, perfumes and fragrances, and also in the production of some precursors of standard antioxidants such as *p*-cresol.

The oxygenated monoterpenes accounted for 3.56–7.01% for umbels and stems, respectively. In total, three oxygenated monoterpenes were identified. The major compound of this fraction was terpinen-4-ol (3.25–6.74% for umbels and stems, respectively). However, *(E)*-verbenol (0.31%) was detected only in umbel EOs.

Sesquiterpene hydrocarbons were 1.8–4.5% for stems and umbels, respectively. *α*-copaene, bicyclogermacrene, germacrene D and *δ*-cadinene were the compounds representing this fraction.

Oxygenated sesquiterpenes were represented by three compounds, with a total ranging between 4.17 and 5.61% for umbels and stems, respectively. The major component of this fraction was spathulenol, which varied between 2.56 and 3.74%.

**Figure 3:**
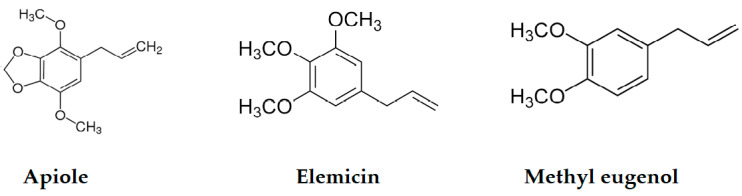
Phenylpropanoid derivatives of *D. tortuosa* EOs.

Three phenylpropanoid derivatives, apiole, methyl eugenol and elemicin, were detected in low amounts (Figure 3). Apiole, a characteristic metabolite of the family Apiaceae, was detected only in umbels EOs.

**Figure 4:**
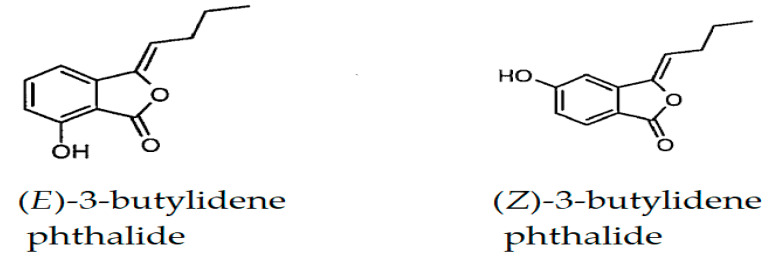
Phtalide derivatives of *D. tortuosa* EOs.

Finally, two phthalide derivatives were detected. Their chemical structures are represented in Figure 4. 

*(E)*-3-Butylidene phthalide was detected at 3.67% in umbel EO and only 0.86% in stem EO. *(Z)*-3-butylidene phthalide was detected only in umbels (0.29%). 

The composition reported proves the variability in the EOs from stems and umbels. Phthalide have been found mainly in plants from the Apiaceae family, such as *Apium graveolens* L., *Angelica sinensis* (Oliv.) Diels, *A. acutiloba* (Siebold & Zucc.) Kitag., *Levisticum officinale* W.D.J. Koch, *Cnidium officinale* Makino and *Ligusticum porteri* J.M.Coult. & Rose [43,44,45,46]. The presence of this group of compounds confers to the Apiaceae family several biological activities, as phthalides are a class of secondary metabolites with a wide range of pharmacological and agronomical activities.

It is known that the production of EOs and secondary metabolites varies depending on the pedoclimatic conditions in the first instance and also on the collection and extraction conditions. The variability of production according to pedoclimatic conditions and the origin of the plant material is called ecological chemistry. The latter is a source of biodiversity and a richness in active molecules synthesized by plants following interactions with their biotopes. Ecological chemistry contributes significantly to the discovery and bioinspiration for the search for active molecules. In the case of *D. tortuosa*, a great variability in compounds and chemotypes has been described in the literature [21,22,34,35,36,37,47]. Table 2 reports the literature data on the chemical variability of *D. tortuosa* EOs.

According to the literature, the chemical composition of *D. tortuosa* EOs has been described for plants growing in Tunisia, Libya, Egypt and Algeria. Different chemotypes have been described, and the EOs varied depending on the area, season, plant part, and condition (fresh/dry) of the plant material [21,22,34,35,36,37,47]. The variability in yield and chemical composition reflects the interaction and the close relationship between the production of molecules and their ecological environment.

### 2.2. Antioxidant Activity

**Figure 5:**
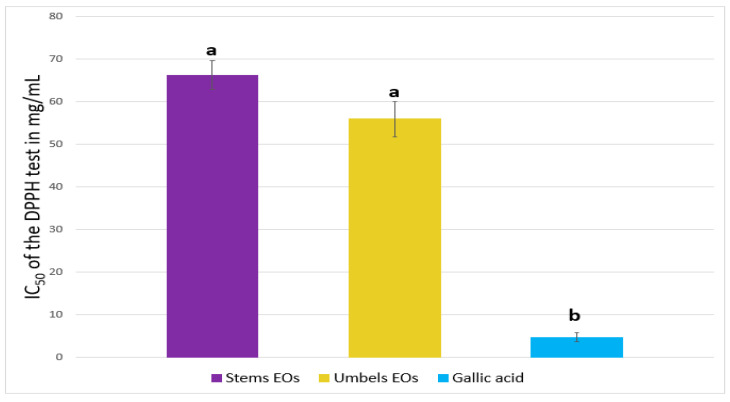
IC_50_ of the EOs expressed as IC_50_ (mg/mL). Means followed by the same letter are not significantly different according to multivariate analysis ANOVA (*p* ≤ 0.05). Data are the mean of three replicates, and ±bars indicate the standard deviation of the mean.

The antioxidant potential of the EOs was evaluated by two free radical scavenging methods. The first method is the DPPH test since it makes it possible to measure the antiradical power of pure molecules or plant extracts in a model system. It measures the ability of an antioxidant to reduce the chemical radical DPPH (2,2-diphenyl-1-picrylhydrazyl) by hydrogen transfer. The EOs were evaluated with reference to the antioxidant potential of gallic acid. The results are expressed as IC_50_ (μL of EO/mL of DPPH solution). The results obtained are shown in Figure 5.

Both EOs showed a potential for trapping DPPH with IC_50_ values of 66.21 and 55.87 mg/mL for stem and umbel, respectively. On the other hand, this activity was moderated by comparing it with the activity of gallic acid.

**Figure 6:**
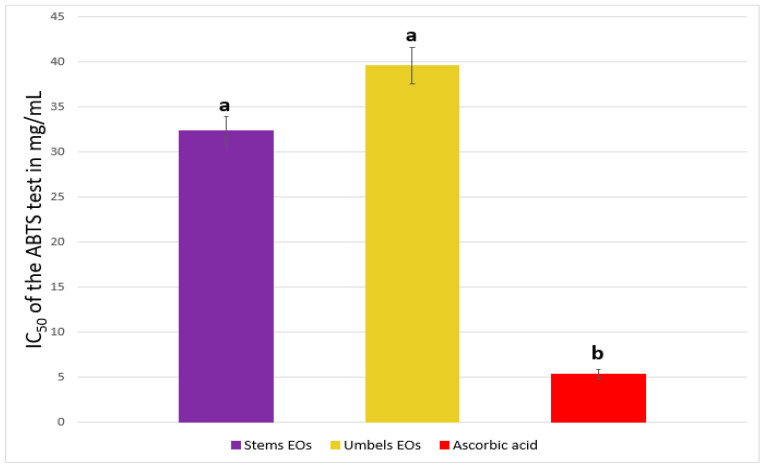
IC_50_ (mg/mL) of the EOs (ABTS test). Means followed by the same letter are not significantly different according to multivariate analysis ANOVA (*p* ≤ 0.05). Data are the mean of three replicates, and ± bars indicate the standard deviation of the mean.

The radical scavenging activity was also evaluated by the ABTS (2,2′-azino-bis-(3-ethylbenzothiazolin-6-sulfonic acid) cation radical decolorization test. The main results are shown in Figure 6.

Both oils showed antioxidant potential for scavenging ABTS radicals, with IC_50_ values of 32.36 and 39.57 mg/mL for stem and umbel EOs, respectively. These activities were significant but moderate in comparison with ascorbic acid (IC_50_ = 5.35 mg/mL).

The Apiaceae family is rich in antioxidant metabolites. The antioxidant activities of EOs and crude extracts have been described in the literature for *Foeniculum vulgare* Miller [11], *Anegelica acutiloba* [48], *Daucus carota* L. [49], and even *D. tortuosa* [35]. On the other hand, the antioxidant potential observed in this study can be related to the chemical composition, which showed a richness in monoterpenes, sesquiterpenes, some phenylpropanoids and phthalides. All these compounds are known for their antioxidant activities [50]. According to the literature, the antioxidant potential of monoterpenes, including the main constituents of *D tortuosa* EOs, such as *α*-pinene, sabinene and *cis*-ocimene, has been reported [51]. Apiole, elemicin and methyl eugenol have also been reported for their antioxidant activities as well as phthalide derivatives [48].

### 2.3. Phytotoxic Activity

**Figure 7:**
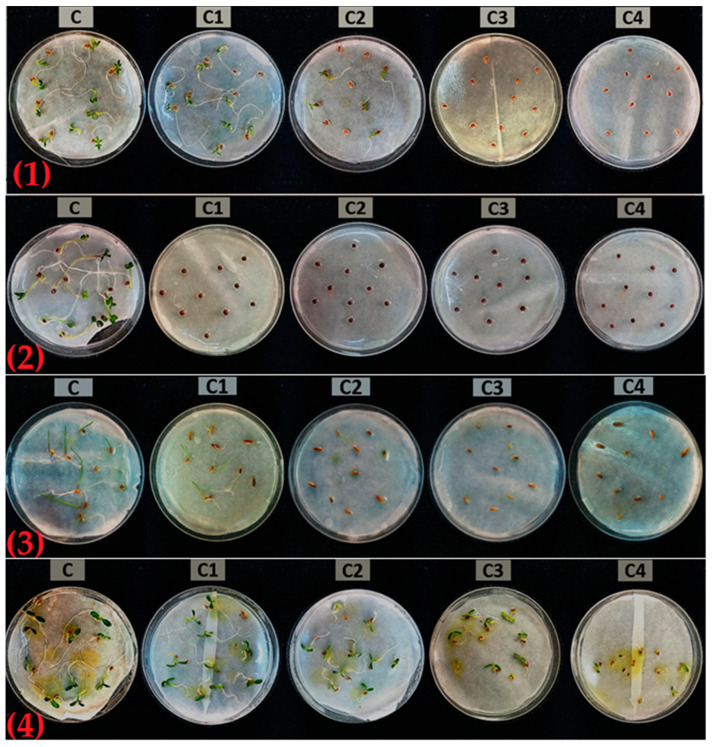
Inhibitory effects of the EOs on germination of tested seeds. (**1**): *L. sativum*, (**2**): *S. arvensis*, (**3**): *L. rigidum*, (**4**): *T. campestre*. C = 0 mg/mL, C1 = 1 mg/mL, C2 = 2 mg/mL, C3 = 3 mg/mL, C4 = 4 mg/mL.

In the current study, *D. tortuosa* EOs have been tested for their herbicidal potential against the germination and growth of weed dicots (*S. arvensis* and *T. campestre*) and monocots (*L. rigidum*) and also against the crop, *L. sativum* (Figure 7), and phytotoxicity was compared to the synthetic herbicide, glyphosate. The results are shown in Table 3, Table 4 and Table 5.

The EOs showed significant effects on the germination and growth of the aerial and root parts of the tested plants.

Both EOs significantly inhibited the germination of all tested seeds in a dose-dependent manner. At 1 mg/mL, the germination of *T. campestre* was around 100%, and no significant effects on the inhibition of germination either by *D. tortuosa* EOs or glyphosate were detected. *T. campestre* was more sensitive to stem EO with a total inhibition at 3 mg/mL. At the same dose, the inhibition was 33% with umbel EO and 43% with glyphosate.

A total inhibition of germination of *L. sativum* was obtained at 3 mg/mL for both EOs, whereas glyphosate determined a partial inhibition (33%).

*S. arvensis* and *L. rigidum* were the most sensitive to the action of the Eos. At 2 mg/mL, germination was totally inhibited, as with glyphosate.

These results show a significant anti-germinative activity of the Eos, which often exceeds that of glyphosate. The inhibition results varied depending on the tested seed and also the EO.

The EOs were phytotoxic not only in the inhibition of germination but also in the growth of the aerial parts and roots when compared to the control. The EOs inhibited the growth of shoots of tested species with a dose–response effect (Table 4). Both EOs, at 1 mg/mL, inhibited by 50% the growth of shoots of *T. campestre*, which germination was not affected by the same dose. *S. arvensis*, also resistant to germination inhibition, resulted in total inhibition at 1 mg/mL. At the same dose, the shoot length of *L. rigidum* was inhibited by 72.8 and 77.1% by the EOs of stems and umbels, respectively. At 2 mg/mL, the shoot length of *L. sativum* was severely inhibited by 61.5 and 72.1%, respectively, by stem and umbel EOs. The inhibitory potency of the EOs on shoot growth was similar to that of glyphosate.

The inhibitory activity of both EOs was also significant on radical growth (Table 5). At 2 mg/mL, *L. sativum* root length was inhibited by 72.8 and 88.5%, respectively, by the EOs of stems and umbels, and the radical length of *T. campestre* showed inhibition of 88.5 and 89.5%, respectively. The stem and umbel EOs, at 1 mg/mL, provoked inhibition of root leghth of *L. rigidum* by 62.7 and 73.1%, respectively. 

It has been proven that even if some seeds of the test species were able to germinate, they were successively inhibited in both shoot and root length in a combined phytotoxicity event.

These results agree with the literature. In fact, Eos and aqueous and organic extracts (petroleum ether, acetone and chloroform) from the aerial parts of *D. tortuosa* were evaluated for their allelopathic potential against two cultivated species, *Brassica rapa* L. and *Linum usitatissimum* L., showing a remarkable herbicidal potential [32]. The EOs contain high amounts of monoterpene hydrocarbons (81.05 and 81.84 for stems and umbels, respectively). This class of terpenes is known for its herbicidal potential and its involvement in the allelopathic potential of several plants. The results obtained in this study prove the close relationship between the richness in monoterpene hydrocarbons in *D. tortuosa* EOs, neglecting the possible interactions of synergism and antagonism of the different components detected in the two EOs [52,53,54,55,56]. 

Sabinene, present in appreciable amounts in the EOs (16.23–18.67%), has been reported for its herbicidal properties, with inhibition of the growth of shoots and roots of *Poa annua* L. and *Amaranthus retroflexus* [57]. Moreover, the EO of a sabinene-rich chemotype of *Ravensara aromatica* Sonn. was reported to have herbicidal properties against the germination and growth of garden cress (*Lepidium sativum* L.) and rice (*Oryza sativa* L.) [58].

The phytotoxicity of *α*-phellandrene, among the major components of the EOs (6.3–11.74%), was described by De Martino et al. (2010) [52]. This compound inhibited germination and root growth of *Raphanus sativus* and *Lepidium sativum* [52]. Similarly, other components of the EOs of *D. tortuosa* have been reported for their phytotoxic potential, in particular, *β*-pinene, *β*-myrcene, *α*-terpinene, *p*-cymene, *γ*-terpinene and *α*-terpineol [52,56].

Several studies showed that the involvement of EOs and/or their pure components in the phenomenon of allelopathy is linked to the induction of oxidative stress, leading to the alteration of the vital functions of plants [56]. Furthermore, some EOs have been shown to inhibit germination and growth following spray application, provoking an alteration of membrane integrity via the peroxidation of fatty acids from membrane phospholipids, resulting in the release of malondialdehyde, blocking chlorophyll synthesis, inducing proline accumulation and resulting in plant desiccation [53].

*α*- and *β*-pinene, in appreciable amounts in the EOs reported in this research, have been reported to induce physiological effects resulting in the inhibition of germination and growth of several weeds [54,55,56]. Indeed, the application of these two compounds on seeds of *Cassia occidentalis* L. and *Oryza sativa* induced an inhibition of chlorophyll synthesis and an increase in the content of macromolecules such as proteins and carbohydrates, accompanied by an inhibition of activity of some hydrolyzing enzymes such as proteases, *α*-amylases and *β*-amylases. On the other hand, the activity of peroxidases, superoxide dismutase, guaiacol peroxidase, catalase, ascorbate reductase and polyphenol oxidases increased, reflecting the oxidative stress generated by the application of these molecules [54,56]. Similarly, pinenes have been reported to disrupt energy metabolism on roots of maize seedling by inhibiting oxidative phosphorylation and the electron transport chain [50]. All of these results prove the importance of essential oils of *D. tortuosa* as candidate molecules for allelopathic applications in sustainable agriculture.

### 2.4. Antifungal Activity of D. tortuosa EOs

**Figure 8:**
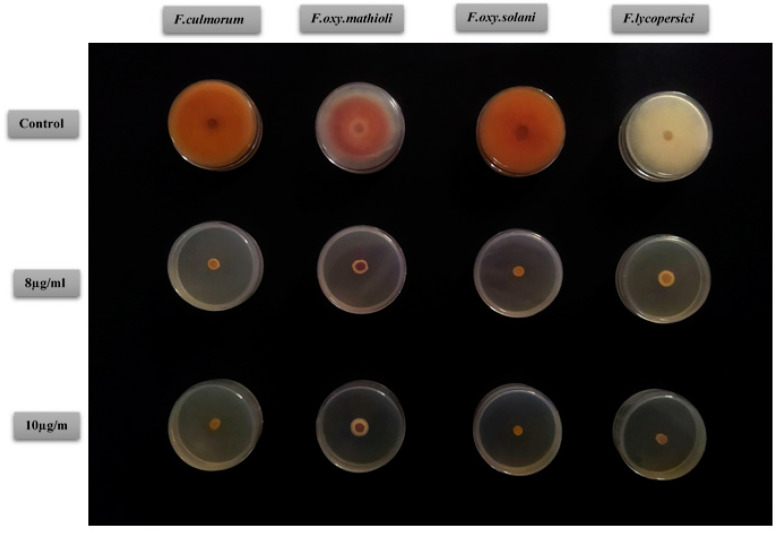
Inhibitory effects of the EOs from stems and umbels on the mycelial growth of four phytopathogenic fungal strains.

EOs exhibiting interesting antifungal activities depending on the tested strains, EOs were screened at different concentrations. Statistical analysis revealed a significant difference concerning the concentration of each oil and also significant differences among the inhibitory effects of the EOs. Similarly, strains of phytopathogens reacted in different ways to the action of the EOs (Figure 8). The results are shown in Table 6.

The umbel EOs showed the most significant effects compared to the stems. A total inhibition was obtained at the dose of 8 mg/mL against *F. oxysporum solani* and at a dose of 10 mg/mL for the other strains tested, and these two doses were retained as MIC. However, the inhibition by the stem EO, even at 10 mg/mL, was always partial. *F. lycopersici* and *F. oxysporum mathioli* were the two most resistant strains to the action of stem EO.

The results obtained confirm the antimicrobial potential of *D. tortuosa* EOs, which have been reported on bacteria and other strains of phytopathogenic fungi. In fact, Krifa et al. [32] reported a total inhibition of the growth of *Fusarium graminearum* and *Alternaria* sp. at 6 mg/mL of *D. tortuosa* EO.

Sabinene, one of the major components of *D. tortuosa* EOs, has been described as having bactericidal activity, and its inhibitory effects on biofilm formation and pH tolerance have been reported [59].

Phthalide derivatives, detected in our study in the EOs, are known for their antifungal potential. These molecules, in particular 3-*n*-butylidenephthalide, are known to have antifungal potential that often exceeds that of synthetic fungicides such as itraconazole, fluconazole and ketoconazole [60,61,62]. The presence of these compounds could contribute to the activity of *D. tortuosa* Eos and also explains the difference in activity between umbel and stem EOs. 

The antifungal activity of EOs can be attributed to the effect of secondary metabolites synthesized by plants in the context of their allelopathic interactions with the different components of their biotopes. This antimicrobial effect depends on the substances with antifungal properties present in each plant and each biotope. 

## 3. Materials and Methods

### 3.1. Plant Material

Aerial parts of *Deverra tortuosa* (stems and umbels) were harvested from the Djerissa region located in the northwest of Tunisia, characterized by a semi-arid bioclimatic stage. Five different samples from different plants separated by at least 50 m were collected and then dried in a glass greenhouse for 10 days; then, the plant material was used for the extraction of the EOs. For the herbicidal assays, mature seeds of *Trifolium campestre* Schreb, *Sinapis arvensis* L., *Lepidium sativum* L. and *Lolium rigidum* Gaudin were collected from crop fields (Table 7).

Voucher specimens of collected plants (Table 7) were identified by Professor Hamrouni Lamia and deposited at the Laboratory of Genetic and Forest Ecology of the National Research Institute of Rural Engineering, Water and Forests, Tunisia.

### 3.2. Extraction of the Essential Oils

The EOs were extracted using a Clevenger-type apparatus for four hours. The EOs were weighed for yield calculation and then dried over anhydrous sodium sulfate and stored in sealed glass bottles at 4 °C. The yield was calculated based on the dry weight of samples by the following formula:

$$\mathrm{Oil}\;\mathrm{yield}\;(\%,\;W/W)\;=\;\frac{weight\;of\;essential\;oils\;(g)}{dried\;weight\;of\;plant\;material\;(g)}*100$$

### 3.3. Gas Chromatography Analysis with MS Detection

The GC-MS analysis of the EOs was carried out on an Agilent 6890 gas chromatograph equipped with a fused silica capillary column HP-5MS (30 m × 0.25 mm film, 5% phenylmethyl-siloxane and 0.25 μm). The EOs were dissolved in *n*-hexane (2 mg/mL). The injection of 1 μL of each sample was carried out in split mode with a ratio of 1:100; helium was used as the carrier gas at a rate of 1 mL/min. The operative conditions were as follows: temperature of injector 250 °C, temperature of detector 280 °C. The oven temperature varied as follows: 35 °C to 325 °C with a rate of 5 °C/min. The equilibration time was 0.5 min. MS operate in electron impact was at a potential of 70 eV in a scan range from 50–550 m/z. The identification of the components was based on their Kovats retention indices and their comparison with those of the literature [38,39,40,41], and their mass spectra with those available in the NIST 02 and Wiley 275 mass spectral libraries [63]. The Kovats indices were calculated related to a series of *n*-alkanes (C8–C30). This identification of some components was confirmed by co-injection with authentic compounds: *α*-pinene, sabinene, *α*-phellandrene, *α*-terpinolene, methyl eugenol and *β*-phellandrene (purchased from Sigma-Aldrich, Germany). Quantification was electronically recorded from FID area data using ChromCard program, Thermoquest.

### 3.4. 2,2 -Diphenyl-1-picrylhydrazyl (DPPH) Assay

The free radical scavenging activity was assessed using the DPPH assay, according to the method described by Ud-Daula et al. (2016) [64]. A total of 300 µL of samples, EOs or butylated hydroxytoluene (purchased from Sigma-Aldrich, Germany) used as standard at different concentrations or methanol (blank), were added to 2 mL of freshly prepared DPPH (purchased from Sigma-Aldrich, Darmstadt, Germany) methanolic solution (0.1 mM). The mixture was vortexed and incubated at room temperature for 30 min in the dark, and the absorbance was then measured at 517 nm against a blank on a Biowave IIWPA spectrophotometer (Serlabo Technologies). The antiradical activity was expressed as IC_50_ (mg/mL), the amount of sample required to scavenge 50% of free radicals present in the test solution. All experiments were conducted in triplicate, and data are expressed as mean values ± SD

### 3.5. ABTS^•+^ Free Radical Scavenging Activity

The ABTS^•+^ radical assay was performed according to procedures reported by Ud-Daula et al. [64]. A solution containing ABTS^•+^ radical cations (ABTS^+^) was prepared by mixing an equal volume of potassium persulfate (2.45 mM) and ABTS (7 mM) (purchased from Sigma-Aldrich, Darmstadt, Germany). The mixture was then incubated for 16 h at room temperature in the dark. The final obtained solution was then diluted with ethanol to an absorbance of 0.70 at 734 nm. A total of 200 µL of various concentration of tested EOs (5–100 mg/mL) and ascorbic acid (used as a standard) were mixed with 2 mL of the ABTS radical solution and allowed to incubate in the dark at room temperature for 5 min. Then, the absorbance was measured at 734 nm on a Biowave IIWPA spectrophotometer (Serlabo Technologies). The inhibition percentage was calculated against the blank. All experiments were conducted in triplicate; results are expressed as IC_50,_ the concentration of tested sample needed to scavenge 50% of ABTS radical cation.

### 3.6. Herbicidal Activity of Deverra tortuosa Eos

Seeds of *Sinapis arvensis*, *Trifolium campestre*, *Lepidium sativum* and *Lolium rigidum* were used in phytotoxic activity assays. Before germination tests, seeds were disinfected with 5% sodium hypochlorite. Ten seeds were placed in Petri dishes lined with double-layer filter paper Whatman No.1 and treated with different doses (1, 2, 3, 4 mg/mL) of the EOs in a solution of Tween 20 (0.1%) [65]. The herbicidal potential of the EOs was compared to the activity of the synthetic herbicide, glyphosate. Phytotoxic assays were performed in a completely randomized design, and each assay was replicated three times. After 10 days, the germination percentages were calculated, and the growth of roots and shoots was measured in cm.

### 3.7. Antifungal Activity

#### 3.7.1. Fungal Strains

Four phytopathogenic fungal strains were used. *F. oxysporum solani* was obtained from the laboratory of plant protection of the Tunisian National Institute of Agronomic Research, and *Fusarium oxysporum* sp. *lycopersici*, *F. oxysporum* mathioli and *F. culmorum* were obtained from Dr Hirsch’s Laboratory, University of California Los Angeles (UCLA).

#### 3.7.2. In Vitro Antifungal Activities on Mycelial Growth

The antifungal properties of the EOs were tested in vitro in Petri dishes using the agar dilution method [66]. The samples were diluted in a Tween 20 solution (0.1% *v*/*v*) and then added to 10 mL of PDA at 40 °C to provide the required concentrations (6, 8 and 10 µL/mL). PDA plates containing only Tween 20 (0.1%) were used as the negative control. A mycelial disk of 6 mm in diameter was placed in each PDA plate, then incubated at 24 °C for 7 days. All assays were repeated with three replicates for each strain and each dose. The fungicidal properties were determined as the percentage of inhibition (PI) of mycelia growth compared to the control, following the formula:

$$PI.\%=\frac{\left(dc-dt\right)}{dt}\times 100$$

where dc and dt are the mean diameter of control growth and treated fungi, respectively. Minimum inhibitory concentration is determined as the lowest dose at which there is no fungal growth.

### 3.8. Statistical Analysis

EOs yield, antioxidants tests, germination and seedling growth experiments and antifungal assays were carried out using a randomized block design and three replications for each assay. Statistical analyses were performed with Statistical Package for the Social Sciences (SPSS version 23.0. IBM Corp.: Armonk, NY, USA). Results were examined statistically using one-way analysis of variance (ANOVA) followed by Student–Newman–Keuls tests. The differences between individual means were considered significant at *p* ≤ 0.05.

## 4. Conclusions

Plants always contain a mixture of several biological molecules that act simultanously or differently, take similar or different paths, and act together or independently on one or more targets, thus leading to an important biological potential, contributing to an organic and producible agriculture, healthy environment, and also a rich and sustainable ecosystem. In this sense, the EOs of *Deverra tortuosa* showed a richness and diversity of compounds. These oils vary depending on the part of the plant (umbels or stems), the same with the literature that is related to the origin of the plant, the season of collection and the drying of the plant material. This makes this species a source of various metabolites with various interests, above all, that in this current study, several biological activities have been demonstrated. Indeed, the antioxidant, antimicrobial and also herbicidal potential have been proven during this study, which may suggest a possible use of *D. toruosa* EOs in sustainable agricultural practices, both as herbicides and antifungals. Furthermore, these EOs can form the basis of environmentally friendly formulations. 

## Figures and Tables

**Table 1:** Composition of EOs from stems and umbels of *D. tortuosa*.

Compound ^a^	LRI _Exp_ ^b^	LRI _Lit_ ^c^ [38,39,40,41]	Area%		Identification ^d^
			Umbels	Stems	
α-Thujene	926	930	4.20	2.37	MS, RI
***α*** **-Pinene**	**935**	**939**	**24.2**	**28.88**	MS, RI, Inj
**Sabinene**	**971**	**975**	**16.23**	**18.67**	MS, RI, Inj
***β*** **-Pinene**	**976**	**979**	**5.10**	**6.20**	MS, RI
*β*-Myrcene	992	990	2.64	2.50	MS, RI
***α*** **-Phellandrene**	**1003**	**1002**	**11.74**	**6.30**	MS, RI, Inj
*α*-Terpinene	1014	1017	1.07	1.42	MS, RI
***p*** **-Cymene**	**1021**	**1024**	**-**	**6.77**	MS, RI
***β*** **-Phellandrene**	**1027**	**1029**	**6.70**	**-**	MS, RI, Inj
***cis*** **-Ocimene**	**1034**	**1037**	**7.85**	**5.28**	MS, RI
*γ*-Terpinene	1057	1059	1.33	2.17	MS, RI
*cis*-Sabinene hydrate	1073	1070	-	0.27	MS, RI
*α*-Terpinolene	1085	1088	0.35	0.49	MS, RI, Inj
*neo-allo*-Ocimene	1146	1144	0.43	-	MS, RI
*trans*-Verbenol	1147	1144	0.31		MS, RI
**Terpinen-4-ol**	**1174**	**1177**	**3.25**	**6.74**	MS, RI
*α*-Copaene	1372	1376	0.18	-	MS, RI
Methyl eugenol	1406	1403	0.55	0.81	MS, RI, Inj
Germacrene D	1480	1485	1.99	0.77	MS, RI
Bicyclogermacrene	1502	1500	1.72	0.58	MS, RI
*δ*-Cadinene	1525	1523	0.61	0.45	MS, RI
Elemicin	1554	1557	0.36	-	MS, RI
Spathulenol	1573	1578	2.56	3.74	MS, RI
*β*-Eudesmol	1651	1650	0.65	0.73	MS, RI
*α*-Vadinol	1656	1654	0.96	1.14	MS, RI
*(E)-3*-**Butylidene phthalide**	1675	1672	0.29	-	MS, RI
Apiole	1680	1678	0.55	-	MS, RI
*(Z)-3-* ***Butylidene phthalide***	1721	1718	3.67	0.86	MS, RI
Total			99.49	97.14	
Monoterpene hydrocarbons			81.84	81.05	
Oxygenated monoterpenes			3.56	7.01	
Sesquiterpene hydrocarbons			4.5	1.8	
Oxygenated sesquiterpenes			4.17	5.61	
Phenylpropanoids			1.46	0.81	
Phthalide derivatives			3.96	0.86	

^a^ = Components are listed in their order of elution from a HP-5 MS column, and their percentages were calculated from a flame ionization detector (FID); ^b^ = calculated linear retention index relative to a series of *n*-alkanes (C8–C30) on a HP-5MS column; ^c^ = linear retention index relative to the literature data [38,39,40,41]; ^d^ = identification method; RI: retention index; MS = mass spectrometry; Inj = co-injection with authentic compounds; compounds higher than 5.0% are highlighted in boldface; - = absent.

**Table 2:** Chemical diversity of EOs extracted from *D. tortuosa* according to the literature.

	Tunisia [22]		Tunisia [37]		Tunisia [36]				Lybia [35]		Algeria [21]	Egypt [34]	Egypt [47]
	Fresh Plant	Dry Plant	November	April	Stems		Seeds		Summer	Spring	Aerial Parts	Aerial Parts	Aerial Parts
					Origin 1	Origin 2	Origin 1	Origin 2					
*α*-Thujene	-	-	1.2	0.6	2.8	1.5	2.1	1.9	6.64	21.22	1.2	0.44	
*α*-Pinene	6.2	5.1	5.5	1	11.0	8.9	32.2	37.7	-	3.30	11.4	-	15
*α*-Fenchene	-	-	0.06	-	-	-	-	-	-	8.45	-	-	
Camphene	-	-	0.06	-	-	0.2	0.3	0.5	-	-	0.2	-	33
Sabinene	38.9	35.8	11.0	-	-	2.4	4.1	3.2	-	2.04	6.4	18.49	
*β*-Pinene	1.8	2.1	1.7	-	-	6.5	27.8	30.7	-	-	3.9	-	
*β*-Myrcene	8.9	12.5	1.1	-	-	6.9	3.4	3.2	-	1.30	1	18.81	
1,8-Cineole	-	-	-	-	-	-	-	-	13.43	4.42	-	-	15.2
Linalool	-	-	-	-	-	0.2	-	0.3	-	27.9	-	-	
*α* -Phellandrene	0.8	1.8	0.9	-	-	7.2	-	0.6	-	-	8.3	-	
Δ-3-carene	2.9	1.9	0.2	0.08	-	-	7.8	3.0	-	-	0.1	-	
*α*-Terpinene	-	-	0.7	0.6	0.7	0.3	5.9	0.3	-	-	0.2	-	
*p*-Cymene	4.9	8.8	7.0	2.2	5.8	8.7	0.9	1.3	-	-	2.6	1.17	
Limonene	4.3	5.0	10.9	4.1		-	-	-	-	-	15.8	-	
*β*-Phellandrene	1.3	1	-	-	17.2	-	-	-	-	-	3.2	-	
*cis*-Ocimene	5.6	6.6	1.9	0.7	0.7	0.6	0.8	1.4	-	-	0.4	1.53	
Borneol											-		16.6
Myrtenol	-	-	26.2	0.3	-	-	-	-	-	0.38	-	-	
Terpinen-4-ol	-	-	1.8	39.6	1.7	1.1	0.2	1.2	-	-	0.9	8.09	
Cubebene	-	-	-	-	-	-	-	-	2.62	10.9	0.1	-	
Aromadendrene	-	-	0.2	0.2	-	-	-	-	6.25	8.41	0.1	-	
Myristicin											27.4	0.89	
*β*-Caryophyllene	-	-	-	-	-	-	-	-	11.62	-		-	
Elemicin	-	-	-	-	-	-	-	-	-	-	-	12.9	
*α*.-Cadinol	tr	0.7	-	-	-	-	-	-	21.51	-	0.2	-	
*(Z)-3*-Butylidene phthalide	5.0	1.5	2.4	4.1	-	-	-	-	-	-	-	-	
*(E)-3*-Butylidene phthalide	-	-	5.9	11.4	-	-	-	-	-	-	-	-	

Origin 1: Bougharara; Origin 2: Naffatia; -: not detected.

**Table 3:** Effects of EOs on seed germination (%).

	Doses	% Germination		
	(mg/mL)	Stem EOs	Umbel EOs	Glyphosate
*T. campestre*	0	100 ± 0 ^A^	100 ± 0 ^A^	100 ± 0 ^A^
	1	100 ± 0 ^Aa^	100 ± 0 ^Aa^	100 ± 0 ^Aa^
	2	66.66 ± 15.27 ^Ba^	73.33 ± 5.77 ^Ba^	63.33 ± 5.77 ^Bb^
	3	0 ± 0 ^Cc^	33.33 ± 5.77 ^Cb^	43.33 ± 5.77 ^Ca^
	4	0 ± 0 ^Cb^	20 ± 0 ^Da^	23.33 ± 5.77 ^Da^
*L. sativum*	0	100 ± 0 ^A^	100 ± 0 ^A^	100 ± 0 ^A^
	1	90 ± 10 ^Ba^	96.66 ± 5.77 ^Ba^	90 ± 0 ^Ba^
	2	53.33 ± 5.77 ^Cb^	23.33 ± 5.77 ^Cc^	80 ± 10 ^Ca^
	3	0 ± 0 ^Db^	0 ± 0 ^Db^	33.33 ± 10 ^Da^
	4	0 ± 0 ^Da^	0 ± 0 ^Da^	0 ± 0 ^Ea^
*L. rigidum*	0	96.66 ± 5.77 ^A^	96.66 ± 5.77 ^A^	96.66 ± 5.77 ^A^
	1	50 ± 10 ^Ba^	33.33 ± 5.77 ^Bb^	13.33 ± 5.77 ^Bc^
	2	0 ± 0 ^Ca^	0 ± 0 ^Ca^	0 ± 0 ^Ca^
	3	0 ± 0 ^Ca^	0 ± 0 ^Ca^	0 ± 0 ^Ca^
	4	0 ± 0 ^Ca^	0 ± 0 ^Ca^	0 ± 0 ^Ca^
*S. arvensis*	0	86.66 ± 15.27 ^A^	86.66 ± 15.27 ^A^	86.66 ± 15.27 ^A^
	1	30 ± 10 ^Ba^	23.33 ± 5.77 ^Bb^	13.33 ± 5.77 ^Bc^
	2	0 ± 0 ^Ca^	0 ± 0 ^Ca^	0 ± 0 ^Ca^
	3	0 ± 0 ^Ca^	0 ± 0 ^Ca^	0 ± 0 ^Ca^
	4	0 ± 0 ^Ca^	0 ± 0 ^Ca^	0 ± 0 ^Ca^

Data are the mean of three replicates ± SD. Within each species, different capital letters in the same column indicate that means are different among concentrations, and different lowercase letters in the same line compare the activity of EOs and glyphosate for the same species and the same dose according to multivariate analysis ANOVA (*p* ≤ 0.05).

**Table 4:** Inhibitory effects of EOs on the length of aerial parts.

Herbs	Doses (mg/mL)	Shoot Length (cm)		
		Stem EOs	Umbel EOs	Glyphosate
*L. sativum*	0	2.08 ± 0.07 ^Aa^	2.08 ± 0.07 ^Aa^	2.08 ± 0.07 ^Aa^
	1	1.19 ± 0.09 ^ABa^	1.25 ± 0.06 ^ABa^	2.29 ± 0.11 ^Ab^
	2	0.8 ± 0.47 ^ABa^	0.58 ± 0.07 ^ABa^	0.26 ± 0.02 ^Ba^
	3	0 ± 0 ^Ba^	0 ± 0 ^Ba^	0 ± 0 ^Ba^
	4	0 ± 0 ^Ba^	0 ± 0 ^Ba^	0 ± 0 ^Ba^
*L. rigidum*	0	3.76 ± 0.9 ^Aa^	3.76 ± 0.9 ^Aa^	3.76 ± 0.9 ^Aa^
	1	1.02 ± 0.07 ^Ba^	0.86 ± 0.11 ^Bab^	0.76 ± 0.11 ^Bb^
	2	0 ± 0 ^Ba^	0 ± 0 ^Ba^	0 ± 0 ^Ba^
	3	0 ± 0 ^Ba^	0 ± 0 ^Ba^	0 ± 0 ^Ba^
	4	0 ± 0 ^Ba^	0 ± 0 ^Ba^	0 ± 0 ^Ba^
*S. arvensis*	0	1.72 ± 0.28 ^Aa^	1.72 ± 0.28 ^Aa^	1.72 ± 0.28 ^Aa^
	1	0 ± 0 ^Ba^	0 ± 0 ^Ba^	1.7 ± 0.05 ^Ab^
	2	0 ± 0 ^Ba^	0 ± 0 ^Ba^	0.28 ± 0.01 ^Bb^
	3	0 ± 0 ^Ba^	0 ± 0 ^Ba^	0 ± 0 ^Ba^
	4	0 ± 0 ^Ba^	0 ± 0 ^Ba^	0 ± 0 ^Ba^
*T. campestre*	0	1.4 ± 0.93 ^Aa^	1.4 ± 0.93 ^Aa^	1.4 ± 0.93 ^Aa^
	1	0.7 ± 0.08 ^ABa^	0.77 ± 0.07 ^ABa^	1.3 ± 0.08 ^Ab^
	2	0.54 ± 0.05 ^ABa^	0.52 ± 0.1 ^ABa^	0.31 ± 0.1 ^Ba^
	3	0 ± 0 ^Ba^	0.5 ± 0 ^ABb^	0.28 ± 0.01 ^Bc^
	4	0 ± 0 ^Ba^	0.25 ± 0.08 ^Ba^	0 ± 0 ^Ba^

Data are the mean of three replicates ± SD. Within each species, different capital letters in the same column indicate that means are different among concentrations, and different lowercase letters in the same line compare the activity of EOs and glyphosate for the same species and the same dose according to multivariate analysis ANOVA (*p* ≤ 0.05).

**Table 5:** Inhibitory effects of EOs on radical growth.

Herbs	Doses (mg/mL)	Roots Length in cm		
		Stem EOs	Umbel EOs	Glyphosate
*L.sativum*	0	5.31 ± 1.08 ^Aa^	5.31 ± 1.08 ^Aa^	5.31 ± 1.08 ^Aa^
	1	3.7 ± 0.3 ^ABa^	1.25 ± 0.23 ^ABc^	2.57 ± 0.41 ^ABb^
	2	0.95 ± 0.14 ^Ba^	0.61 ± 0.12 ^Bb^	0.66 ± 0.02 ^Bb^
	3	0 ± 0 ^Bb^	0 ± 0 ^Bb^	0.35 ± 0.06 ^Ba^
	4	0 ± 0 ^Ba^	0 ± 0 ^Ba^	0 ± 0 ^Ba^
*L.rigidum*	0	3.49 ± 1.6 ^Aa^	3.49 ± 1.6 ^Aa^	3.49 ± 1.6 ^Aa^
	1	1.3 ± 0.2 ^Bbc^	0.94 ± 0.10 ^Ba^	1.68 ± 0.43 ^Bc^
	2	0 ± 0 ^Ba^	0 ± 0 ^Ba^	0 ± 0 ^Ba^
	3	0 ± 0 ^Ba^	0 ± 0 ^Ba^	0 ± 0 ^Ba^
	4	0 ± 0 ^Ba^	0 ± 0 ^Ba^	0 ± 0 ^Ba^
*S. arvensis*	0	2.83 ± 1.19 ^Aa^	2.83 ± 1.19 ^Aa^	2.83 ± 1.19 ^Aa^
	1	0 ± 0 ^Bb^	0 ± 0 ^Bb^	0.43 ± 0.06 ^Ba^
	2	0 ± 0 ^Bb^	0 ± 0 ^Bb^	0.29 ± 0.01 ^Ba^
	3	0 ± 0 ^Ba^	0 ± 0 ^Ba^	0 ± 0 ^Ba^
	4	0 ± 0 ^Ba^	0 ± 0 ^Ba^	0 ± 0 ^Ba^
*T. campestre*	0	4.25 ± 0.64 ^Aa^	4.25 ± 0.64 ^Aa^	4.25 ± 0.64 ^Aa^
	1	1.32 ± 0.08 ^ABb^	0.94 ± 0.07 ^Bc^	2.85 ± 0.08 ^Aa^
	2	0.49 ± 0.7 ^Ba^	0.45 ± 0.18 ^Ba^	0.54 ± 0.11 ^Ba^
	3	0 ± 0 ^Bb^	0.5 ± 0 ^Ba^	0.38 ± 0.02 ^Ba^
	4	0 ± 0 ^Ba^	0.33 ± 0.08 ^Ba^	0 ± 0 ^Ba^

Data are the mean of three replicates ± SD. Within each species, different capital letters in the same column indicate that means are different among concentrations, and different lowercase letters in the same line compare the activity of EOs and glyphosate for the same species and the same dose according to multivariate analysis ANOVA (*p* ≤ 0.05).

**Table 6:** Percent inhibition of the EOs on the mycelia growth of four *Fusarium* strains.

	Fungal Strains Growth Inhibition (%)							
	*F. culmorum*		*F. oxysporum mathioli*		*F. oxysporum * *solani*		*F. lycopersici*	
Doses	Umbels	Stems	Umbels	Stems	Umbels	Stems	Umbels	Stems
6 mg/mL	94.55 ^a^ ± 2.35	71.42 ^a^ ± 7.06	86.99 ^a^ ± 2.81	43.08 ^b^ ± 10.15	91.83 ^b^ ± 4.08	75.51 ^b^ ± 4.08	82.22 ^c^ ± 4.44	49.62 ^b^ ± 2.56
8 mg/mL	95.91 ^a^ ± 4.08	76.87 ^a^ ± 2.35	91.86 ^a^ ± 2.81	57.72 ^ab^ ± 7.45	100 ± 0.0 ^a^	83.67 ^a^ ± 4.08	92.59 ^b^ ± 2.56	55.55 ^ab^ ± 4.44
10 mg/mL	100 ± 0.0 ^a^	83.67 ^a^ ± 4.08	95.12 ^a^ ± 4.87	62.6 ^a^ ± 2.81	100 ± 0.0 ^a^	87.75 ^a^ ± 4.08	100 ± 0.0 ^a^	60 ^a^ ± 4.44
MIC (mg/mL)	10	>10	>10	>10	8	>10	10	>10

Data are the mean of three replicates ± SD. Within each fungi species, different letters in the same column indicate that means are different among concentrations according to multivariate analysis ANOVA (*p* ≤ 0.05).

**Table 7:** Origins, voucher specimen number, used parts and date of collect of plant material.

Collected Species	Used Parts	Preserved Specimens	Date of Harvest	Origins
*Deverra tortuosa*	Umbels	DIJ0122	September 2022	Djerissa, Kef
	Stems	DSJ0122	September 2022	Djerissa, Kef
*Lepidium sativum* L.	Seeds	LS22	June 2022	Kalâat el-Andalous, Ariana
*Lolium rigidum* Gaudin		LR22	July 2022	Sidi ismail, Beja
*Sinapis arvensis* L.		SA22	July 2022	Sidi ismail, Beja
*Trifolium campestre* Schreb.		TC22	July 2022	Sidi ismail, Beja

## Data Availability

All data are available in the manuscript file.
